# BIG DATA … small story

**DOI:** 10.1136/jech-2020-215752

**Published:** 2020-10-28

**Authors:** Bernard C K Choi

**Affiliations:** 1 Public Health Agency of Canada, Ottawa, Canada; 2 University of Toronto, Toronto, Canada; 3 University of Ottawa, Ottawa, Canada

**Keywords:** Public health, Record linkage, Registers, Research methods, Vision

## BIG DATA: WHAT IT IS

In modern society, a huge volume of data (big data) are being collected and accumulated, and growing exponentially.^1 2^ Data come from the internet, social media, medical records, customer databases, massive open data and other data sources. Big data are huge in quantity (volume), fast in production sometimes in real time (velocity), structured and unstructured in data types (variety), inconsistent and changing (variability), uncertain in data quality (veracity) and potentially useful (value).^3 4^

Big data, when analysed properly, can improve corporate performance, increase productivity, provide insights and predict future scenarios for better decisions in medicine, public health, science and technology, business and other sectors.^5 6^ Big data, when misused, can lead to data breach and privacy concerns^6^ and, when misinterpreted^7^ or manipulated,^8^ can cause false predictions leading to potentially disastrous consequences.

## SMALL STORY: PIZZA DELIVERY

Here is a small story that might help understand the impacts of big data. It is in part inspired by some ideas already posted on the internet.^9 10^ The story is about a customer who orders pizza by phone, and the interesting exchange that ensues, delightful and not so delightful, that potentially can happen during the big data era.

Pizza : *[The phone rings]* Thanks for calling. How may I help you?

Customer : I would like to order a pizza.

Pizza : What is your customer number?

Customer : XY1357

Pizza : Hi, Mr. Lee. Your address is 789 Main Street. Your phone number is 987–1234. Which credit card do you want to use—the one ending in 1122, or 3210, or 2468?

Customer : How do you know my credit cards?

Pizza : We are connected to the CRM system—Customer Relationship Management. You want the usual?

Customer : You know my usual?

Pizza : According to our records, you ordered pizza with pepperoni and mushrooms nine times in the last 2 months.

Customer : I want a seafood pizza this time.

Pizza : Seafood is not good for you.

Customer : Why?

Pizza : According to your medical records, you have high cholesterol and high blood pressure.

Customer : What would you recommend?

Pizza : You should try our new low-fat healthy pizza.

Customer : Are you sure I will like it?

Pizza : Last week you borrowed a cookbook from the Central Library on ‘Low-Fat Healthy Recipes’.

Customer : OK, I will order a large ‘low-fat healthy pizza’. Is it big enough for five people?

Pizza : It is big enough for your family. Facebook tells us that you have a spouse and two boys aged 6 and 9. Your mother also lives with you. Please make sure that she keeps her portion small. We see she had heart bypass surgery done just last month.

Customer : OK, OK. Can I pay by credit card now?

Pizza : Let me check your credit cards... Sorry, you have to pay cash. All three of your credit cards have exceeded the credit limits.

Customer : OK, I will go get some cash at a bank machine.

Pizza : According to the records, you have already exceeded your daily withdrawal limit for today.

Customer : Then deliver the pizza to my home. I have cash at home.

Pizza : Don’t worry about the money. We can directly access your bank account, where you have direct paycheque deposit. This account is the best.

Customer : How long will it take for the delivery?

Pizza : About 30 min. Or you can pick it up. It is faster.

Customer : Why?

Pizza : According to our GPS—Global Positioning System—you are in a car right now on Main Street making a right turn onto First Avenue. It will take you 4 min and 32 s to get here.

Customer : This is excessive and awful! It seems like you are spying on me every moment.

Pizza : I’m sorry. But we are doing this just to help you!

Customer : What?! *[hangs up]*

## BE PREPARED FOR THE BIG DATA ERA

The short story on pizza delivery is rooted in reality, perhaps a bit exaggerated at this time of our technology, but is totally feasible because the big data era advances so rapidly. This thought-provoking story exposes some potential benefits and issues of big data. There are questions that are tough to answer. When you phone a stranger, would you like that person to know every detail of your life? Would you like your whereabouts being tracked in real time over your life? What if your personal information stored in big data has been modified? Meal delivery based on customers’ food preferences may improve customer service. Linking eating habit information with medical data may improve a person’s lifestyle behaviour for a longer and better life. But when life choices are dictated by automated computer algorithms, life becomes mechanical and loses the human touch. Financial, such as personal banking, information as part of big data can facilitate money transfer and fraud prevention but, on the other hand, can be hacked and misused.

Should big data stop at some line to give people some personal space? Who would decide where that line should be, and enforce and ensure there are consequences when the line is crossed? It is a dilemma. On the one hand, big data are felt to be doing the tracing and tracking of the society to help people, livelihood and the economy. On the other hand, big data are felt to be spying on the society. A big issue for big data is, while individuals’ information is collected for big data, it is difficult if not impossible for individuals to ask big data for their own individual information, or even know the extent to which their individual information is being collected and stored in big data.

The big data era is here whether we like it or not. So keep thinking, and keep asking questions, before big data get too big and out of control.
